# Training the next-generation of biomedical scientists through artificial intelligence-driven education and research in pharmacology and pharmaceutical sciences

**DOI:** 10.3389/ebm.2026.10988

**Published:** 2026-04-22

**Authors:** Santosh Kumar, Ritu Karwasra, Weinan Zhou, Jayaraman Seetharaman, Bhupesh Singla

**Affiliations:** 1 Department of Pharmaceutical Sciences, College of Pharmacy, The University of Tennessee, Health Science Center, Memphis, TN, United States; 2 Clinical Pharmacology, Central Council for Research in Unani Medicine, Ministry of Ayush, New Delhi, India; 3 School of Biological Sciences, Louisiana Tech University, Ruston, LA, United States; 4 Department of Pharmacology, Addiction Science, and Toxicology, College of Medicine, The University of Tennessee, Health Science Center, Memphis, TN, United States

**Keywords:** artificial intelligence, disease prediction, drug discovery, drug repurposing, machine learning, pharmaceutical sciences, pharmacology, pharmacometrics

## Abstract

Artificial intelligence (AI)-driven graduate education and research in pharmacology and pharmaceutical sciences (AIPPS) aims to address the rapidly-growing role of AI and machine learning (ML) applications in biomedical sciences. This review provides perspectives on why and how the next-generation of biomedical scientists equip themselves with skills necessary to integrate AI and ML tools into their current fields of study, particularly pharmacology and pharmaceutical sciences. The AI-enabled approaches discussed in this article highlight opportunities for improving competitiveness in an evolving scientific landscape, that includes academia, pharmaceutical and biotech industries and regulatory science. Furthermore, this review discusses how graduate education and research can be enhanced through training in AI-driven disease prediction, molecular target identification drug design and discovery, drug repurposing and pharmacometric modelling. The knowledge outlined here may help graduate students and early career researchers navigate the challenges associated with applying AI-based methodologies in fundamental research, product and process development, service delivery, and regulatory policy and ethics. Overall, the insights provided in the review aim to support the development of skilled forward-thinking biomedical and pharmaceutical scientists capable of leveraging AI technologies in modern research environments.

## Impact statement

The information provided in this review is expected to advance AI integration in pharmacology and pharmaceutical sciences, which will help develop novel drugs in a faster manner or repurpose existing drugs for new treatments. The fundamental knowledge and its application discussed here will guide graduate students and early career researchers in AI-driven biomedical research, fostering innovation and enhancing their ability to address complex challenges in disease prediction, target discovery, and drug development. The discussion in this review will also enhance the competitiveness of graduates in scientific marketplaces, including academia and industries, shaping future leaders in biomedical sciences.

## Introduction

The discussion on artificial intelligence (AI)-driven education and research, particularly in the pharmaceutical sciences, comes at the right time as AI-aided applications in every aspect of life are growing exponentially. The widespread adoption of AI-enabled tools has generated significant demand for professionals with strong digital, computational, and analytical competencies. The skills required to be scientists are rapidly evolving as AI becomes increasingly integrated into research and healthcare practices. AI is defined as a machine-based system that can, for a given set of human-defined objectives, make predictions, recommendations or decisions influencing real or virtual environments by the National Artificial Intelligence Initiative Act of 2020. Machine learning (ML) is a key subset of AI that emphasizes the use of statistical algorithms enabling computers to learn patterns from data and improve performance without being explicitly programmed for every task. Deep learning, a further subset of ML, employs multi-layered neural networks and is particularly prevalent in biomedical and pharmaceutical applications due to its ability to handle high-dimensional, unstructured data such as images, sequences, and multi-omics datasets.

Pharmaceutical industries, hospitals, and academic research institutions increasingly seek professionals who apply AI and ML to design drug formulations, clinical and real-world data analyses, regulatory decision support, and other transitional workflows [1]. Further, the Food and Drug Administration (FDA), National Institutes of Health (NIH), and major pharmaceutical companies are investing heavily in AI for drug discovery, drug repurposing, pharmacometrics, pharmacovigilance, precision dosing, advanced analytics, and identification of novel biomarkers for diseases [1]. Despite these trends, there is a significant skill gap between traditional pharmaceutical training and modern AI-enabled research and practices [2]. There is a critical need to bridge this gap by providing focused training in AI tools, methods, and applications relevant to pharmaceutical sciences and research [2]. Therefore, graduate educational offerings in AI at various universities and institutions should evolve to match the changing needs of the job market and hone AI-based skillsets of pharmaceutical and biomedical scientists to increase their competitiveness and competency in the modern high-tech era [3].

**FIGURE 1:**
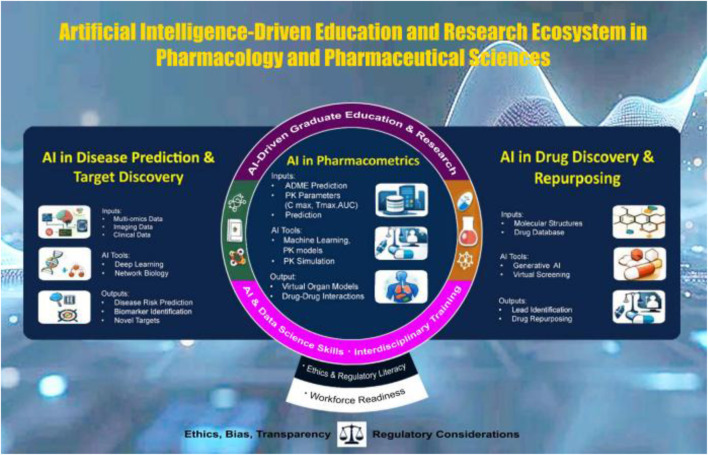
A schematic of AI-driven methodologies and applications in Pharmacology and Pharmaceutical Sciences. The three pillars for graduate research and training include: (1) AI in disease prediction and target discovery (AI-DPTD), (2) AI in drug discovery and repurposing (AI-DDR), and (3) AI in pharmacometrics (AI-PM). Input data types, analytical tools (including machine learning models), and example outputs are illustrated. Acronyms: ADME = absorption, distribution, metabolism, excretion; Cmax = maximum concentration; Tmax = time to maximum concentration; AUC = area under the curve; PK = pharmacokinetics; PD = pharmacodynamics; EHR = electronic health records.

There are several universities in the United States that offer graduate certificate or master’s level training in AI and ML and their potential use in pharmacology, pharmaceutical, biomedical, or biological sciences (Table 1). The existing programs vary in scope and emphasis. Although these certificate and MS programs are great initiatives in the right direction at the right time, more universities and institutions should take the challenge of integrating AI into pharmaceutical and biomedical sciences [3]. Since covering all dimensions of AI/ML-powered biomedical or health sciences is beyond the scope of this review, we discuss three major areas of pharmacology and pharmaceutical sciences in which graduate education and research may be focused to prepare our next-generation of educators and researchers (Figure 1). 1) AI in Disease Prediction and Target Discovery, which explores the transformative role of AI in predicting the progression of human diseases, understanding underlying molecular mechanisms, and identifying viable drug targets, 2) AI in Drug Discovery and Repurposing, focusing on rapidly evolving applications of AI across drug discovery and drug repurposing, and 3) AI in Pharmacometrics, which explores how AI and ML transform pharmacometrics, including pharmacokinetic/pharmacodynamic (PK/PD) modeling, dose optimization, and clinical trial simulation. These educational and research programs may also be expanded to address regulatory and ethical considerations, as well as current limitations of AI in these areas (Figure 1).

**TABLE 1:** Representative master’s or graduate certificate programs on AI and ML in pharmacology, pharmaceutical, biomedical, or biological sciences.

Institution	Program Name	Key Features
University of Florida	MS in artificial intelligence in biomedical and health sciences	Interdisciplinary master’s training AI for biomedical research, clinical care, and healthcare systems
University of Texas at Dallas	MS in artificial intelligence for biomedical sciences	Intersects AI, statistics, and biomedical sciences for research and healthcare applications
University of California, San Francisco	MS in artificial intelligence and computational drug discovery and development	Focus on applying AI/ML to pharmaceutical drug discovery and development
University of Alabama at Birmingham	MS in AI in medicine	Concentrates on application of AI technologies to medical innovation, diagnostics, and clinical systems
Washington University in St. Louis	MS in biomedical data Science and AI	Combines data science with AI techniques aimed at biomedical/clinical research
Northeast Ohio Medical University	MS in health data Science and AI	Health-oriented program blending advanced analytics, AI and clinical data science
University of Louisville	MS in artificial intelligence in medicine	Interdisciplinary master’s focused on AI, machine learning, medical imaging, and healthcare data analysis
UT Health San Antonio	MD/MSAI dual degree (doctor of medicine and master of Science in artificial intelligence)	Combined program where students earn an MD alongside an MS in artificial intelligence, with applications in healthcare (suitable for future AI-focused clinicians/researchers)
University of Maryland	MS in AI for drug development	AI and machine learning for pharmaceutical innovation and drug development
University of Pittsburgh	MS in computational biomedicine and biotechnology	Focuses on AI/ML, genomics, bioinformatics
Drexel University	MS in bioinformatics	Focuses on bioinformatics with ML, data analysis
University of Illinois Urbana-Champaign	MS in bioinformatics	Focuses on data science with ML emphasis
Johns Hopkins University	MS in bioinformatics	Focuses on genomic and computational analysis
Carnegie Mellon University	MS in computational biology and bioinformatics	Computational biology program with ML/data science components
University of North Carolina at Charlotte	MS in bioinformatics	An interdisciplinary program at the intersection of the disciplines of biology, chemistry, mathematics and statistics, computing and informatics and engineering
North Carolina State University	MS in bioinformatics	Combines computer science, statistics, and biology
University of California Santa Cruz	MS in biomolecular engineering and bioinformatics	Focuses on the application of engineering principles to molecular biology and bioinformatics
UTHealth Houston	MS in biomedical informatics	Focuses on data and AI for biomedical research
University of Tennessee, Knoxville	Applied artificial intelligence and medicine certificate	Focuses on diagnostics, treatment planning, and predictive analytics
University of Florida	Artificial intelligence in pharmacy graduate certificate	Focuses on AI and machine learning applications in drug development and informatics
Brandeis University	Master’s certificate in bioinformatics data engineering and AI/ML	Covers bioinformatics workflows, AI/ML analysis of biological data
Washington University in St. Louis	Graduate certificate in biomedical data Science and AI	AI applications in biomedical informatics, biostatistics, data analysis
Ohio State University	Artificial intelligence in digital health certificate	Focuses on AI’s role in medicine and healthcare analytics
University of Alabama at Birmingham	AI in medicine graduate certificate	Credential teaching foundational AI methods and clinical healthcare applications
Johns Hopkins University	AI in healthcare professional certificate	Focuses on applying AI concepts to clinical and healthcare systems
Michigan Technological University	Artificial intelligence in healthcare graduate certificate	Blends AI, machine learning, clinical data modeling, and healthcare technology analysis
Rutgers University	AI in healthcare certificate	Focuses on AI implementation, data science, and improving health care delivery
Old Dominion University	AI in healthcare graduate certificate	Combines AI foundations with healthcare informatics applications (machine learning, explainable AI)
Brandeis University	Bioinformatics data engineering and AI/ML certificate	Covers ML and deep learning applied to bioinformatics/biology
Stanford University	Biomedical data Science graduate certificate	Focuses on data science methods for biomedical data
Ohio State University	Biomedical informatics graduate certificate	Focuses on health analytics and informatics skills
University of South Dakota	Bioinformatics graduate certificate	Focuses on AI tools and bioinformatics

## Enhancing disease prediction and target discovery through AI-based models

AI is rapidly reshaping biomedical research by transforming how disease progression is predicted, mechanisms are deciphered, and therapeutic targets are identified [4]. Complex human diseases, including Alzheimer’s disease (AD), stroke, cancers, and various metabolic and cardiovascular pathologies, are driven by intricate interactions across genomic, molecular, cellular, physiological, and environmental factors [4]. Traditional hypothesis-driven research approaches are often insufficient to manage the multidimensional complexity of modern biomedical data, including diverse circulating proteins, cleaved receptors, cytokines, and the spatial distribution of various proteins/genes across different tissues and organs in both healthy and diseased conditions [5]. In this context, AI has emerged to efficiently manage data complexity and offer faster mechanistic understanding [5]. Preparing graduate students and researchers to work at this intersection should, therefore, be a critical educational and research priority.

AI-driven disease prediction and target discovery (AI-DPTD) can train the next-generation of biomedical scientists to integrate computational domains with systems biology and translational medicine [6]. By combining molecular disease mechanisms, multi-omics analytics, graph- and deep-learning architectures, and real-world clinical datasets, this approach will equip students to accelerate the prediction of various disease development processes and identify novel druggable targets [6]. This approach seems highly beneficial for complex and comorbid diseases, including HIV comorbidities with stroke, AD, substance use disorders, cardiovascular diseases, infectious diseases, and cancers [4]. For instance, AI and ML models are promising tools to analyze complex and multimodal clinical data improving infectious disease surveillance, diagnosis, treatment, and outcome prediction [7]. Through AI-enabled approaches, students will learn why classical statistical parametric approaches are insufficient for modeling high-dimensional data characterized by nonlinearity, missingness, and interaction effects [8]. The students will learn to apply supervised, unsupervised, reinforcement, and deep learning paradigms for analytical strategies [8]. This integrated approach will help students develop computational knowledge and intellectual rigor to predict whether the generative model is meaningful, robust, and clinically applicable.

A major pillar of AI-DPTD is hands-on engagement with biomedical data, in which students will be trained to integrate genomic, transcriptomic, proteomic, metabolomic, epigenomic, and imaging data, as well as electronic health records (EHR) [9]. Through this integration, students will discover various limitations and gain practical skills for data cleaning, preprocessing, and integration using multi-omics fusion strategies by developing AI/ML frameworks [9]. Through this process, students will learn both classical approaches to disease risk modeling and ML frameworks, including neural networks [9]. Through computational learning/practice sessions and case studies, students will get training to implement these methods to real disease conditions.

With regards to understanding underlying mechanisms and novel target discovery for various diseases, students will examine how AI methods can reconstruct gene regulatory networks, infer signaling cascades, and detect functional modules [10]. Network pharmacology approaches may also provide the identification of druggable target molecular factors and pathways, revealing how polypharmacology may outperform single-target paradigms [10]. AI-guided biomarker discovery further demonstrates how early detection signatures can help improve disease prevention and early treatment in high-risk populations [11]. Early disease prediction and novel target discovery have the potential to shape AI-driven pharmaceutical research, particularly computational AI-based drug designing, repurposing, pharmacometrics, and personalized medicine [2].

AI-DPTD may also integrate ethical, regulatory, and translational perspectives. Students will learn identifying/ruling out bias in datasets that may perpetuate healthcare inequities, explore concerns regarding privacy and federated data use, and consider regulatory challenges in deploying AI tools in clinical settings [12].

Therefore, AI-DPTD possesses the potential to transform graduate education and research, which is a perfect blend of biology, computation, and clinical insight. Such programs will generate scientists capable of leading interdisciplinary teams, communicating across professional boundaries, and translating discoveries into patient benefits with improved efficacy, reduced toxicities, and equitable therapies.

## Accelerating drug discovery and repurposing using the AI-based strategies

The pharmaceutical science research is entering a new era where AI-driven therapeutic innovation is changing the field of drug discovery and development [13]. AI-enabled drug discovery approaches have the potential to significantly improve decade-long timelines, high attrition rates, and escalating costs [13]. The use of AI in drug discovery and repurposing for complex and comorbid diseases that reflect deeply interconnected molecular networks can be expedited [14]. The information obtained by AI-based disease prediction and novel target discovery will aid in harnessing the large volume of data to expedite drug discovery and repurposing in a time-efficient manner [13].

AI-driven drug discovery and repurposing (AI-DDR) represents a transformative approach to therapeutic development, spanning early target identification and molecule design, clinical trial optimization, and regulatory decision-making [6]. Traditional drug discovery and development takes over 10 years and costs >$1 billion, making these drugs very expensive and unavailable/unaffordable for large world populations [15]. Drug discovery (typically 2–5 years in modern pipelines) focuses on target identification, hit finding, and lead optimization, while full drug development (historically 10–15+ years and $1–3 billion) encompasses preclinical, clinical phases, and regulatory approval. AI/ML approaches demonstrate the greatest impact in accelerating early discovery phases, potentially reducing timelines from years to months, reducing costs by up to 40% [16]. AI-enabled platforms, such as AlphaFold’s protein structure prediction and industrial successes from BenevolentAI and Insilico Medicine. BenevolentAI employs knowledge graphs and ML to identify novel targets and pathways. Notable efforts include identification of baricitinib for COVID-19 repurposing and several candidates in neurology and inflammation now in Phase I/II as of 2026. Insilico Medicine utilizes generative AI platforms (e.g., Chemistry42, Pharma.AI) to design molecules *de novo*, with ISM001-055 (targeting TNIK for idiopathic pulmonary fibrosis) showing positive Phase IIa results in 2024–2025, including dose-dependent improvement in forced vital capacity (FVC) and favorable safety. Incorporating AI-driven drug discovery into graduate education and research will ensure that students emerge as scientists and are prepared to critically design discovery strategies in a faster manner [3].

AI-DDR begins by re-establishing the fundamental understanding of classical drug development strategies, target identification through lead discovery, optimization, preclinical validation, clinical testing, and the final approval [13]. ML can now outperform classical quantitative structure-activity relationship (QSAR) methods for predicting potency, selectivity, solubility, stability, and toxicity, significantly reducing attrition caused by poor PK or safety profiles [17]. Moreover, machine learning models such as variational autoencoders (VAEs; deep learning-based models capable of generating new data from latent representations) and generative adversarial networks (GANs; originally introduced by Goodfellow et al. in 2014) are routinely integrated into AI-enabled drug discovery workflows for *de novo* molecular generation and property optimization. AI platforms, for example, Variational Autoencoders, Generative Adversarial Networks, and Reinforcement Learning, generate novel molecules and expedite AI-driven drug discovery [18].

The drug discovery research is driven by AI-based methodologies that are transforming the early phase of drug candidate screening. Instead of screening libraries of millions of existing compounds, AI, specifically generative adversarial networks and variational autoencoders, allows the *de novo* design, creating molecule structures from scratch that meet specific property constraints. By integrating chemical synthesis with ML, drug properties (potency, solubility) can be optimized in iterative cycles. This “molecular mindset” combined with machine intelligence allows the exploration of “middle space” between small molecules and biologics [19]. In addition, tools like AlphaFold have solved the protein folding problem, predicting the three-dimensional (3D) structure of proteins using their amino acid sequence. This capability is now standard, allowing researchers to visualize drug targets with atomic precision [19]. Safety failures being a leading cause of drug attrition, AI models are transforming toxicology from an observational science to a predictive one. Now researchers can use Graph Neural Networks and Quantitative Structure-Activity Relationship models to predict toxicity endpoints such as hepatotoxicity or cardiotoxicity [20]. Research training should emphasize creating a “toxicity knowledge graph” that links chemical structures to adverse outcome pathways, helping to understand the mechanistic basis of toxicity.

Another feature of AI-driven drug discovery is to integrate target identification and validation with systems biology approaches. AI models trained on multi-omics and interaction datasets can infer previously unknown disease-causing genes and gene-protein relationships [21]. Graph Neural Networks can be applied to protein-protein and disease-gene interaction networks, enabling identification of new network hubs, synthetic‐lethal relationships, and polypharmacological opportunities [22]. These exercises reinforce a shift from “one drug, one target” strategy to multi-target network modulation, which is essential for tackling multifactorial and comorbid diseases [23].

During the COVID-19 pandemic, AI-enabled platforms rapidly analyzed viral protein structures, performed ultra-large virtual screening of chemical libraries (>billions of compounds) and prioritized repurposed drugs (e.g., baricitinib identified via BenevolentAI knowledge graphs) compressing early hypothesis generation and candidate prioritization from years to weeks/months compared to traditional approaches.

The challenges with data representation can be overcome by AI-driven drug discovery, where students will learn to work with heterogeneous biomedical data such as molecular fingerprints, 3D structures, protein and sequence embeddings, omics datasets, clinical trial registries, and EHRs [24, 25]. In parallel, AI-aided drug discovery can highlight data-centric limitations, e.g., scarcity, batch effects, bias, missingness, and incomplete annotations [26]. This will provide practical insight into why even the most sophisticated models fail without rigorous data curation.

Drug repurposing offers cost-effective and clinically significant opportunities in drug development, enabling the repositioning of FDA-approved or late-stage drugs for new clinical indications [27]. Students may explore to integrate drug-disease-gene networks, repurposing hypotheses using natural language processing from massive biomedical literature and comparing transcriptomic “signature matching” with drug-induced gene expression [28]. For example, for COVID-19 treatment discovery, drug expansion strategies, including antiretroviral drugs and pharmacoenhancer, ritonavir, AI-aided shortening of response timelines from years to months during public health emergencies [29]. In addition to drug repurposing, AI-DDR has the potential for personalized medicine [13]. Concepts such as digital twins highlight the possibility of constructing patient-specific computational models capable of predicting drug response trajectories [30].

Another critical dimension of AI-DDR addresses the clinical trial ecosystem, where AI/ML increasingly drives patient stratification, recruitment optimization, and outcome prediction [31]. ML algorithms segment patient populations to identify responders and minimize heterogeneity, thereby increasing trial power that may require smaller cohorts [32]. Bayesian ML frameworks model survival outcomes and enable adaptive trial designs, dynamically updating dosing or enrollment strategies [33]. Exposure to these applications helps understand the full translational continuum, from molecular insight to regulatory submission, that often lacks conventional methods [34].

While studying AI-DDR, it is crucial to maintain a consistent focus on ethical oversight and regulatory literacy [35]. Students should critically engage with questions surrounding algorithmic bias in chemical and clinical datasets, transparency in black-box model decision-making, intellectual property issues related to AI-generated compounds, and evolving FDA/EMA policies governing computational models in drug development pipelines [36]. Such discussions underscore that future investigators should balance technical innovation with responsibility, credibility, and societal trust.

By bridging computation with molecular therapeutics and embedding real-world translational experiences into training, AI-DDR will equip new generation of scientists to become leaders in pharmaceutical innovations. These scientists will not only accelerate drug discovery and repurposing but will also ensure that AI is applied responsibly, equitably, and with unwavering focus on improving patient outcomes. Through AI-DDR, students can move beyond conceptual mastery into authentic translational research. Using an integrated computational framework (e.g., Graph Neural Network) supported by a range of tools (e.g., RDKit, DeepChem, Scikit-learn), student teams will build QSAR models, generate novel molecules, perform virtual screening, and construct knowledge graphs for drug repurposing, [37]. This experiential learning builds computational fluency, communication skills, and collaborative practices vital for multidisciplinary academic research and pharma industry R&D careers.

## Reimagining pharmacometrics education and research through artificial intelligence

Pharmacometrics (PM) serves as the quantitative backbone of drug development, regulatory decision-making, and clinical dose optimization [38]. Through PK, PD, pharmacogenomics (PGx), and exposure-response modeling, PM integrates biology, mathematics, and pharmacology to translate experimental data into therapeutic strategies [38]. However, the emerging complexity of biomedical data, including high-dimensional omics profiles, longitudinal EHRs, and heterogeneous real-world evidence, exceeds the analytical capabilities of classical Nonlinear Mixed-Effects (NLME) modeling [39].

Nonlinear mixed-effects (NLME) models have long been central to population pharmacokinetic/pharmacodynamic (PK/PD) modeling, dose optimization and clinical trial simulation. However, the increasing availability of heterogeneous, large-scale, multi-modal datasets including genomic, transcriptomic, imaging, real-world evidence and electronic health records presents analytical challenges that traditional parametric approaches like NLME may not optimally address due to nonlinearity, high dimensionality, missingness and complex interactions. AI and ML frameworks offer complementary strengths in handling such data complexity.

AI now offers powerful computational tools that extend, refine, and, to some extent, transform traditional PM workflows [39]. Through AI-driven PM studies, ML-based algorithms integrated with mechanistic modeling can address challenges in translational PM and precision medicine [40].

Traditional PM approaches focus on physiologically motivated differential equations using population-level NLME models implemented in platforms such as NONMEM, Monolix, Phoenix, R, and Python [41]. These frameworks excel at parameter interpretability, incorporating biological constraints, sparse sampling, and regulatory transparency [42]. However, they rely primarily on prespecified model structures and assume relatively simple variance relationships [42]. Real-world patient records contain missing data, complex covariate correlations, nonlinear exposure-response relationships, and deeply nested interactions across genetics, comorbidity, and polypharmacy [43]. AI approaches, particularly Ensemble Methods, Deep Neural Networks, and Hybrid Mechanistic Data-Driven Models can address these challenges by identifying complex, non-linear mappings [44].

Upon establishing basic foundations of PM (PK, PD, and PG) and statistical methods to derive physiological exposure-effects relationships, students can focus on core AI and ML concepts, equipping them with tools to engage directly in advanced analytics. They will master workflows spanning data preprocessing, feature extraction, model building, performance evaluation, and cross-validation. Furthermore, key distinctions between regression, classification, clustering, and dimensionality reduction can be explored in relation to typical PM tasks such as dose prediction, responder classification, covariate stratification, and exposure heterogeneity analysis [2]. For these reasons, hands-on experience with platforms such as Scikit-learn, TensorFlow, PyTorch, and cloud-based resources, connecting biomedical modeling with scalable computational infrastructures are needed [45]. The training will emphasize ML-based PK and PD modeling challenges with diverse data streams (clinical trial datasets, longitudinal EHR, and omics measurements), accompanied by inherent limitations (noise, imbalance, bias, and missingness) [46].

ML methodologies demonstrate utility in predicting primary PK parameters (clearance, volume of distribution, and half-life), especially when datasets encompass extensive covariates, which are difficult to accommodate using classical nonlinear models [47]. Moreover, advanced time-series neural networks, including Recurrent Neural Networks and Attention-Based Frameworks, capture delayed drug effects and dynamic exposure-response processes [48]. For dose optimization, students will learn how ML algorithms predict individualized dosing regimens that minimize drug toxicity while maintaining efficacy, especially for the treatment of brain diseases [49]. Drug-drug interaction predictions provide further illustration of ML’s capacity to learn complex metabolic competition patterns from EHR and post-marketing surveillance data [50].

One of the most impactful intersections in AI-PM emerges through PGx integration. Polymorphisms in genes encoding metabolic enzymes, transporters, and receptors are major determinants of therapeutic response and safety [51]. Traditional PGx analyses rely on stratified subgroup comparisons or covariate inclusion into NLME models [52]. ML approaches, by contrast, permit high-dimensional integration across hundreds of genetic variants simultaneously, revealing gene-gene interaction clusters and predictive signatures that defy univariate testing [53]. Through AI-driven PGx, students can explore population stratification algorithms, genomics-based risk models, and methods for identifying rare but clinically significant outlier responses for precision dosing [54].

AI-PM also addresses AI-driven virtual trials and digital twins. Digital twins are defined as patient-specific computational models that simulate drug exposure and response trajectories, representing a major shift in clinical decision-making [55]. Students can learn how ML-derived phenotype embeddings can augment classical PK/PD frameworks to simulate treatment outcomes within virtual populations [56]. These approaches inform trial design, dosing strategies, and early go/no-go decisions while minimizing unnecessary patient exposure [55]. Virtual bioequivalence studies illustrate ML-enhanced models that simulate comparative plasma profiles across formulations under diverse demographic constraints, potentially reducing the burden of physical bioequivalence trials [57].

As AI becomes embedded in regulatory decision-making, model evaluation and interpretability form another critical pillar of education [58]. Students can address validation strategies required to demonstrate the reliability, robustness, and generalizability of predictive systems to regulatory authorities [58]. The graduate training includes explainable AI techniques such as Feature Importance Ranking, Local Interpretable Model-Agnostic Explanations (LIME), and Shapley Additive Explanations (SHAP) to bridge the transparency gap inherent in neural network models [59]. Students may practice articulating how AI output can be reconciled with established PM standards and regulatory expectations, preparing them for multidisciplinary engagements. Bias in EHR data may reflect unequal access to care, differential disease documentation, and incomplete demographic representation [60]. Students may examine how biased training datasets can propagate inequitable dosing algorithms or inaccurate safety predictions. Privacy concerns surrounding genomic and real-time patient data, as well as accountability issues related to black-box AI recommendations, reinforce the need for responsible technological stewardship [61]. Through this study, the learners cultivate ethical literacy aligned with the societal responsibilities of clinical scientists.

AI-driven PM will create future scientists capable of navigating intersecting domains, such as biology, statistics, AI, clinical medicine, and regulatory science. Graduates and researchers trained under this framework will shape the evolution of model-informed drug development, advance personalized dosing strategies, optimize clinical trials, and contribute to AI-guided regulatory science.

## Discussion

By cultivating capacities within a structured academic environment, AI-driven DPTD, DDR, and PM directly address workforce demands in pharmacology, pharmaceutical sciences, biomedical informatics, public health analytics, and precision medicine enterprises (Figure 1). These studies represent the convergence of biological, pharmacological, and pharmaceutical sciences with AI and translational medicine, which are essential for training modern graduate students and researchers. It will encourage interdisciplinary scientists capable of integrating data analytics with biological insight, navigating regulatory landscapes, and communicating effectively across scientific domains. Therefore, offering AI-driven graduate programs and research opportunities in pharmacology and pharmaceutical sciences can be an exciting platform to train the next-generation of biomedical scientists.
